# Omega-3 fatty acid deficiency disrupts endocytosis, neuritogenesis, and mitochondrial protein pathways in the mouse hippocampus

**DOI:** 10.3389/fgene.2013.00208

**Published:** 2013-10-28

**Authors:** Jane A. English, Akiko Harauma, Melanie Föcking, Kieran Wynne, Caitriona Scaife, Gerard Cagney, Toru Moriguchi, David R. Cotter

**Affiliations:** ^1^Department of Psychiatry, Royal College of Surgeons in Ireland, ERC Beaumont HospitalDublin, Ireland; ^2^Proteome Research Centre, School of Medicine and Medical Science, UCD Conway Institute of Biomolecular and Biomedical Research, University College of DublinDublin, Ireland; ^3^Department of Food and Life Science, Azabu UniversitySagamihara, Japan

**Keywords:** Omega-3, proteomics, neuritogenesis, mitochondrial dysfunction, clathrin mediated endocytosis, schizophrenia

## Abstract

Omega-3 fatty acid (n-3 FA) deficiency is an environmental risk factor for schizophrenia, yet characterization of the consequences of deficiency at the protein level in the brain is limited. We aimed to identify the protein pathways disrupted as a consequence of chronic n-3 deficiency in the hippocampus of mice. Fatty acid analysis of the hippocampus following chronic dietary deficiency revealed a 3-fold decrease (*p* < 0.001) in n-3 FA levels. Label free LC-MS/MS analysis identified and profiled 1008 proteins, of which 114 were observed to be differentially expressed between n-3 deficient and control groups (*n* = 8 per group). The cellular processes that were most implicated were neuritogenesis, endocytosis, and exocytosis, while specific protein pathways that were most significantly dysregulated were mitochondrial dysfunction and clathrin mediated endocytosis (CME). In order to characterize whether these processes and pathways are ones influenced by antipsychotic medication, we used LC-MS/MS to test the differential expression of these 114 proteins in the hippocampus of mice chronically treated with the antipsychotic agent haloperidol. We observed 23 of the 114 proteins to be differentially expressed, 17 of which were altered in the opposite direction to that observed following n-3 deficiency. Overall, our findings point to disturbed synaptic function, neuritogenesis, and mitochondrial function as a consequence of dietary deficiency in n-3 FA. This study greatly aids our understanding of the molecular mechanism by which n-3 deficiency impairs normal brain function, and provides clues as to how n-3 FA exert their therapeutic effect in early psychosis.

## Introduction

Important new evidence indicates that dietary omega-3 fatty acid's (n-3 FA) significantly reduces the risk of progression to psychosis in adolescences with at-risk mental state (ARMS) (Amminger et al., 2010). Other studies have reported reduced n-3 FA in red blood cell membranes, platelets, and in post-mortem brain of patients with schizophrenia (McNamara, 2009), and there is now strong evidence to suggest that n-3 FA deficiency is a preventable risk factor for schizophrenia (McNamara, 2011). Preclinical rodent studies have demonstrated that supplementation with n-3 FA can reverse stress induced behavioral and cognitive abnormalities (Ferraz et al., 2011; Feng et al., 2012), and even protect against MK-801 induced neurotoxicity in the prefrontal cortex (Ozyurt et al., 2007). More recently, Bondi and colleagues have shown that n-3 FA deficiency across consecutive generations in a rat model caused impaired cognitive and motivated behavior in adolescent rats (Bondi et al., 2013). The consequences of dietary n-3 deficiency on molecular pathways within the brain are relatively unexplored at the level of the proteome.

Polyunsaturated n-3 FA, particularly docosahexaenoic acid (DHA), are selectively concentrated within the phospholipid bilayer of cell membrane structures in the brain (e.g., synaptic membranes, vesicles, dendritic membranes, and mitochondria). If unavailable, these essential polyunsaturated FA are replaced by non-essential fatty acids, thus alterations in the membrane microenvironment and fluidity can occur, disrupting neuronal membrane dynamics, as well as receptor, transporter, and neurotransmitter function (Horrobin, 1998). Animal models have previously shown that DHA can modulate gene expression (Kitajka et al., 2002) including enhanced dopamine receptor gene expression following maternal deficiency (Kuperstein et al., 2005). In addition, DHA has been shown to enhance neuritogenesis (Kawakita et al., 2006; Beltz et al., 2007; He et al., 2009), and improve mitochondrial function in *in vivo* and *ex vivo* animal models of neurodegenerative disease (Eckert et al., 2013). In order to fully understand the sub-cellular cellular processes that are altered as a consequence of n-3 FA deficiency, we used a discovery based approach to profile differential protein expression changes within the hippocampus of mice following n-3 FA prenatal and trans-generational deficiency. In addition, given our previous findings implicating the cellular trafficking process of clathrin mediated endocytosis (CME) in schizophrenia (Föcking et al., 2011) and evidence implicating n-3 FA in CME in cell culture (Ben Gedalya et al., 2009), we hypothesized that n-3 deficiency influences CME processes in the brain. Therefore, the aim of this study was twofold; (1) To identify protein pathways disrupted as a consequence of n-3 FA deficiency, and (2) To identify CME related proteins that are disrupted as a consequence of n-3 FA deficiency. Using label free LC-MS/MS quantitative proteomics, our investigation focused on the hippocampus from mice chronically deficient in dietary n-3 fatty acids.

## Materials and methods

### Experimental diets and animals

Mice were fed either an n-3 fatty acid deficient (n-3 Def) or n-3 adequate (n-3 Adq) diet for two generations, as previously described (Harauma and Moriguchi, 2011). The experimental diets were based on the AIN-93G diet recommendations for rodents (Reeves et al., 1993), with modifications of the fat source (flaxseed oil) to achieve the low n-3 fatty acid level required (Table 1). The fat content in both diets was 5% (w/w) diet, and there was no difference in the total n-6 fatty acids between the two diets. The diet was custom prepared at Oriental Yeast, Chiba, Japan.

**Table 1:** **Composition of experimental diets**.

	**Composition (g/100g)**	
	**n-3 Adq.**	**n-3 Def.**
Casein, vitamin free	20	20
Carbohydrate:	65	65
Cornstarch	10	10
α-Cornstarch	15	15
Sucrose	10	10
Glucose	20	20
Dextrose	5	5
Maltose-dextrin	5	5
Cellulose	5	5
Mineral-salt mix	3.5	3.5
Vitamin mix	1	1
L-Cystine	0.3	0.3
Choline bitartrate	0.25	0.25
TBHQ	0.001	0.001
Fat:	5	5
Hydrogenated coconut oil	3.875	4.05
Saffower oil	0.885	0.95
Flaxseed oil	0.24	none
Fatty acid composition^a^		
Saturates	75.1	78.1
Monounsaturates	5.4	4.7
18:2n-6	14.6	14.6
18:3n-3	2.53	0.25
n-6/n-3	5.8	58.5

The two experimental diets, an n-3 fatty acid dequate diet (n-3 Adq) and an n-3 fatty acid deficient diet (n-3 Def), were based on the AIN-93 formulation with several modifications to obtain the extremely low basal level of n-3 fatty acid required in this study.

a The 20:4n-6, 20:5n-3 and 22:6n-3 fatty acids were less than 0.01%, i.e., not detected (nd).

Female CD-1 (ICR) mice were obtained at 3 weeks of age from Charles River Japan, Inc. and fed an n-3 Def or n-3 Adq diet. Animals were maintained within the animal facility under conventional conditions, as previously described (Harauma and Moriguchi, 2011). At 8 weeks of age, they were mated with 9-week-old males of the same strain. Their litters (1st generation) were culled to ten pups and the pups were weaned onto the same diet as their Dams. The male and female offspring (2nd generation) were maintained on each diet. When they were 10 weeks old, brain was collected, and the hippocampus was dissected from the whole brain and frozen at −80°C prior fatty acid analysis. This experimental protocol was approved by the audit committee of Azabu University Animal Experiment, according the guidelines of the Animal Experiment in Azabu University.

To confirm n-3 deficiency in brain tissue, lipid extraction and gas chromatography was carried out as previously described in (Harauma and Moriguchi, 2011), according to the method of Lepage and Roy (1986). Internal standards were used to calculate tissue fatty acid concentration. All data were expressed as the mean ± Standard Error of the Mean (SEM). The fatty acid composition in the hippocampus tissues were evaluated using the Tukey's test after One-Way ANOVA (Statistica, Statsoft Japan, Tokyo, Japan) at *P* < 0.05.

### Sample preparation and label free quantification using LC-MS/MS analysis

For label free LC-MS/MS analysis, the hippocampus of 16 n-3 Def and 16 n-3 Adq samples per dietary group were assessed. Prior to analysis, these 16 samples were sub-pooled according to gender (4 male and 4 female per dietary group) so that 8 × n-3 Def and 8 × n-3 Adq LC-MS/MS runs were performed. Following solubilisation of samples by sonication in triethylammonium bicarbonate (TEAB; Sigma), 50 μg of protein from each of the 8 n-3 Adq and 8n-3 Def samples was denatured for 10 min at 80°C in the presence of 1% RapiGest (Waters). Samples were reduced with TCEP (50 mM) for 60 min at 60°C, followed by alkylation with Iodoacetamide (200 mM) for 30 min in the dark. Digestion with trypsin (Promega; 1μg/μl) was carried out overnight at 37°C, and Tryptic peptides were purified using ZipTips (Millipore) according to the manufacturer's instructions. Samples were resuspended in Solution A (HPLC grade water, 2% acetonitrile, 0.5% acetic acid) where by 1 μg of protein digest (5 μl) was injected per LC-MS/MS run. Each of the 16 samples were run in triplicate, on a Thermo Scientific LTQ ORBITRAP XL mass spectrometer connected to an Dionex Ultimate 3000 (RSLCnano) chromatography system. Each sample was loaded onto Biobasic Picotip Emitter (120 mm length, 75 μm ID) packed in house with Reprocil Pur C18 (1.9 μm) reverse phase media column, and was separated by an increasing acetonitrile gradient, using a 56 min reverse phase gradient at a flow rate of 250 nL/min. From 0 to 7 min 5μl (1μg) of sample was injected and loaded onto the column at a flow rate of 1.5 μl/min. At 7 min the mass spectrometer started to acquire data, the chromatography gradient continued, 7–44 min from 5 to 35% Solution B (acetonitrile containing 3% HPLC grade water and 0.5% acetic acid) at 250 nl/min, 44–50 min from 35 to 90% Solution B at 250 nl/min, 50–55 min at 90% Solution B at 250 nl/min increasing to 900 nl/min, 55–56 min at 90% Solution B to 2% Solution B at 900 nl/min decreasing to 250 nl/min. The mass spectrometer was operated in positive ion mode with a capillary temperature of 200°C, a capillary voltage of 45 V, a tube lens voltage of 100 V and with a potential of 1800 V applied to the frit. Survey full-scan MS spectra (300–2000 Da) were acquired in the Orbitrap with a resolution of 60,000, whereby the FTMS maximum injection time was 700 ms, the Ion trap injection time was 100 ms, and the 7 most intense ions from the preview scan were selected for MS/MS analysis.

### Data analysis

Label-free quantification (LFQ) was performed with Max Quant (V 1.3.0.2) as previously described (Hubner et al., 2010). Methionine oxidations and acetylation of protein N-termini were specified as variable modifications, while carbamidomethylation was specified as a fixed modification, and 2 miscleavages were allowed. Protein and peptide FDR's were set to 0.01. Only proteins with at least two peptides (one uniquely assignable to the protein) were considered as reliably identified. LFQ intensity values were used for protein quantification between groups. Only unique and razor peptides were considered for quantification with a minimum ratio count of. Statistical analyses was performed in Perseus (V 1.3.0.4), whereby the data was log 2 transformed, missing values were replaced by values form the normal distribution, and column normalization was performed by subtracting the median. Student's *t*-test was applied to identify proteins differentially expressed between groups at a 5% threshold, and a permutation-based FDR was applied at a 5% threshold. Ingenuity Pathway Analysis (IPA; www.ingeunity.com) was performed on all statistically significant proteins. The IPA *p*-value was calculated using the right-tailed Fisher's Exact Test and multiple hypothesis correction was based on the Benjamini-Hochberg (B-H) approach at 1% FDR threshold.

### Haloperidol treated mice

In order to assess the effects of psychotropic medication on the expression of the specific candidate proteins identified as altered as a consequence of n-3 deficiency, hippocampal tissue was harvested from C57BL6 mice chronically treated with haloperidol (28 days), as previously described (Föcking et al., 2011). Ethical approval (application no.175) was granted by the Royal College of Surgeons in Ireland Research Ethics Committee. The tissue was processed and analyzed by LC-MS/MS as above.

## Results

### Animals and fatty acid analysis of brain tissue

The diets produced no significant differences in body weight gain between dietary groups. In the fatty acid composition analysis of the hippocampus, total n-3 fatty acids were significantly decreased in the n-3 Def group (*p* < 0.001), in each gender (Table 2). This decrease is mostly attributed the reduced levels of docosahexaenoic acid (DHA; 22:6n-3; Table 2). Saturated and monounsaturated fatty acid were approximately the same level. It is noteworthy that the levels of omega-6 fatty acids (n-6 FA) such 22:4n-6 and 22:5n-6 are considerably increased in the n-3 Def mice. It is well-documented that in the absence of n-3 fatty acids, the brain replaces them with other long chain fatty acids such as 22:4n-6 and 22:5n-6, and these findings are in keeping with previous fatty acid composition analyses from n-3 FA deficient mice (Moriguchi et al., 2000; Harauma and Moriguchi, 2011).

**Table 2:** **Fatty acid composition of the hippocampus**.

**Fatty acid**	**n-3 Def**		**n-3 Adq**	
	**Male (*n* = 5)**	**Female (*n* = 6)**	**Male (*n* = 7)**	**Female (*n* = 6)**
14:0	0.11±0.003	0.12±0.01	0.12±0.01	0.12±0.01
16:0 DMA	2.12±0.04	2.00±0.04	1.98±0.03	1.99±0.02
16:0	19.89±0.30	19.47±0.21	19.27±0.19	19.29±0.32
18:0 DMA	3.38±0.07	3.31±0.06	3.61±0.03	3.59±0.06^**^
18:0	20.80±0.13	20.99±0.03	21.01±0.14	21.64±0.24
20:0	0.28±0.02	0.29±0.02	0.32±0.02	0.30±0.02
22:0	0.44±0.05	0.46±0.03	0.50±0.04	0.46±0.05
23:0	0.11±0.01	0.10±0.01	0.12±0.01	0.10±0.03
24:0	0.61±0.07	0.61±0.06	0.51±0.07	0.40±0.11
Total Sat.	47.73±0.18	47.35±0.19	47.44±0.13	47.90±0.33
16:1n-7	0.21±0.02	0.26±0.01	0.20±0.02	0.20±0.02
18:1 DMA	1.11±0.06	1.18±0.04	1.22±0.05	1.09±0.06
18:1n-7	3.31±0.04	3.34±0.03	3.16±0.03^*^	2.94±0.04^***^^,^^##^
18:1n-9	11.44±0.21	11.76±0.16	12.14±0.17	12.10±0.27
20:1n-9	0.78±0.06	0.82±0.05	0.98±0.09	0.91±0.10
22:1n-9	0.12±0.01	0.15±0.02	0.16±0.02	0.13±0.04
24:1n-9	1.11±0.14	1.19±0.09	1.35±0.15	1.25±0.16
Total Mono.	18.07±0.43	18.71±0.14	19.21±0.23	18.62±0.60
18:2n-6	0.26±0.004	0.28±0.01	0.32±0.01^**^	0.30±0.02
20:2n-6	0.23±0.01	0.17±0.01	0.23±0.01	0.18±0.01
20:3n-6	0.30±0.02	0.25±0.02	0.46±0.03^**^	0.43±0.03^***^
20:4n-6	11.64±0.12	10.63±0.49	10.50±0.14^*^	10.57±0.16
22:2n-6	0.11±0.01	0.11±0.02	0.11±0.01	0.07±0.02^*^
22:4n-6	3.63±0.08	3.37±0.06	2.58±0.09^***^	2.69±0.04^***^
22:5n-6	10.40±0.26	10.30±0.59	0.78±0.05^***^	0.76±0.04^***^
Total n-6 PUFA	26.56±0.30	25.11±0.25^#^	14.92±0.22^***^	15.01±0.18^***^
22:5n-3	0.07±0.003	0.04±0.02	0.16±0.01^*^	0.12±0.03
22:6n-3	4.64±0.04	5.50±0.32	15.46±0.35^***^	15.75±0.34^***^
Total n-3 PUFA	4.71±0.04	5.54±0.32	15.62±0.35^***^	15.88±0.31^***^
Total fatty acids (μg/mg wet wt)	39.11±3.98	34.22±1.45	36.51±2.43	39.48±2.67

Fatty acid methyl esters from 10:0 to 24:1n-9 were analyzed. 10:0, 12:0, 12:1, 14:1, 18:3n-6, 18:3n-3, 20:3n-3, and 20:5n-3 were not detected (nd, i.e., < 0.01%). Each parameter is presented as the mean ± S.E.M.

* P < 0.05,

** P < 0.01,

*** P < 0.001: compared between n-3 Def and n-3 Adq diet group in each gender.

# P < *0.05*,

## P < 0.01: compared between male and female in each diets (One-Way ANOVA and Tukey test.).

### Label free LC-MS/MS quantitative analysis

For label free LC-MS/MS, we assessed the hippocampus of 8 samples per dietary group, including 4 male (sub-pool of 8 mice) and 4 female (sub-pool of 8 mice) in each group. To account for technical variability and reduce the risk of detecting false positives, samples were run in triplicate on the Thermo LTQ Orbitrap. The average MS1 peak width was 30 s, and the scan time ranged between 1 and 2 s, whereby an average of 15 data points were acquired for quantitation (Supplementary Figure S1). Using Max Quant we identified and quantified 1008 proteins for differential expression between the 8 n-3 Def and 8 n-3 Adq samples of the hippocampus. Expression profiling identified 114 proteins as significantly differentially expressed (*p* < 0.05) between the dietary groups (student's *t*-test). Of these, 61 proteins were significantly increased, and 53 proteins were significantly decreased in expression. Six proteins were significant following permutation-based FDR at 5% threshold. Supplementary Table S1 lists the proteins significantly altered as a consequence of dietary n-3 deficiency in the hippocampus, and proteins that remained significant following FDR are marked with an asterisks.

### Pathway analysis

**Figure 1:**
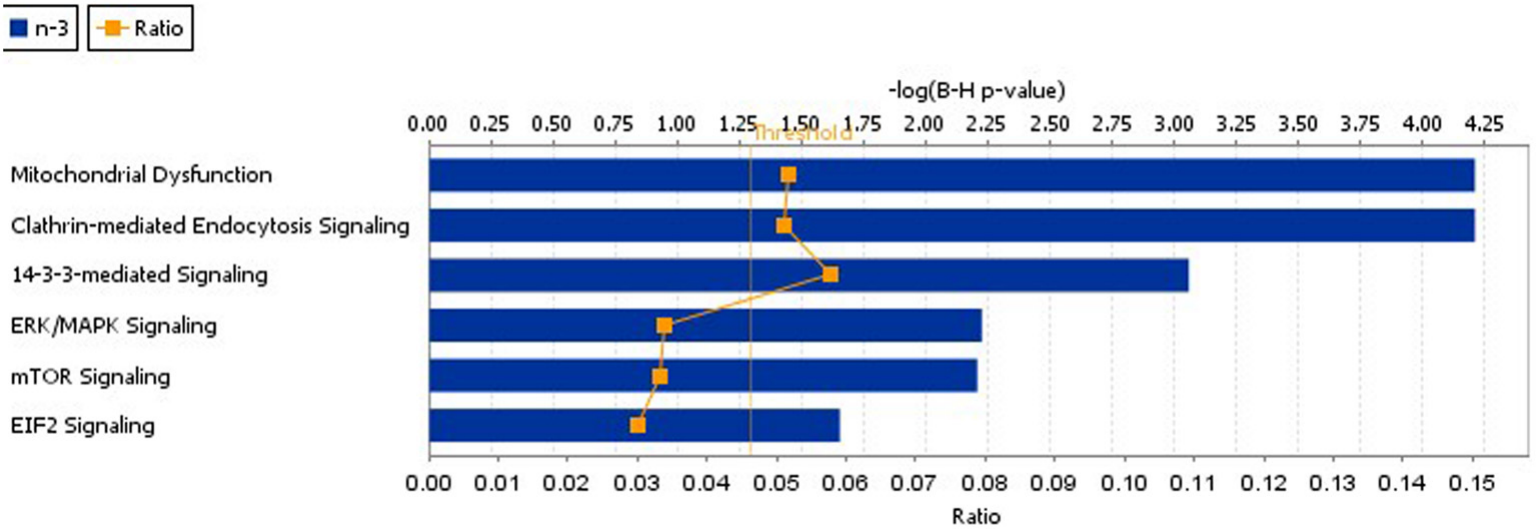
**The top IPA canonical pathways disrupted as a consequence of n-3 FA deficiency.** These pathways included mitochondrial dysfunction, CME, 14-3-3 mediated signaling, ERK,/MAPK signaling, mTor, and EIF2 signaling.

Using IPA we identified cellular function and maintenance as the most significant function disrupted in n-3 FA deficiency (Supplementary Figure S2, Supplementary Table S2). The sub-categories within this process included endocytosis (12 proteins), neuritogenesis (15 proteins), and exocytosis (6 proteins) functions as detailed in Table 3. In addition, IPA reported the top canonical pathways altered as a consequence of n-3 deficiency (Figure 1, Table 4). These were mitochondrial dysfunction (10 proteins), CME (10 proteins), and 14-3-3 signaling (6 proteins) pathways. Canonical pathways within the IPA knowledge have a defined number of molecules, and a ratio to the total number of molecules in the pathway is reported, as well as a B-H *p*-value. IPA identified 49 of the 114 proteins as previously implicated in neurological disease at the mRNA level (Supplementary Table S1).

**Table 3:** **The cellular function and maintenance categories disrupted as a consequence of n-3 FA deficiency in order of significance**.

**Cellular function and maintenance**	***p*-Value**	**Molecules**	**No. Molecules**
Endocytosis	1.79E-04	APOE,ATP5B,CAP1,CLTC,DNM1L,GRB2,HSPA8,MYO5A,PIP5K1C,PPP3CB,RHOB,SH3GL2	12
Neuritogenesis	4.05E-04	ABI2,APOE,CTNNA2,DNM1L,GJA1,MYO5A,PIP5K1C,RAB3A,RALA,RHOB,RTN3,RTN4,SEPT11,STMN1,VAPA	15
Exocytosis	3.89E-02	DNM1L,MYO5A,PIP5K1C,RAB3A,RALA,SRCIN1	6

These sub-categories include endocytosis, neuritogenesis, and exocytosis functions.

**Table 4:** **The top IPA canonical pathways disrupted as a consequence of n-3 FA deficiency**.

**Ingenuity canonical pathways**	**-log(B-H *p*-value)**	**Ratio**	**Molecules**
Mitochondrial dysfunction	4.21E00	10/174 (0.057)	PDHA1,NDUFV1,ATP5B,ATP5A1,OGDH,UQCRC1,
			UQCRC10, NDUFA13,COX4I1,NDUFA8
Clathrin-mediated endocytosis	4.21E00	10/196 (0.051)	HSPA8,APOE,PPP3CB,ARPC5L,GRB2,PIP5K1C,CLTC,SH3GL2,DNM1L,UBC
14-3-3-mediated Signaling	3.06E00	7/121 (0.058)	YWHAQ,TUBA1A,YWHAE,GRB2,YWHAZ,TUBA4A,PRKCE
ERK/MAPK signaling	2.23E00	7/206 (0.034)	YWHAQ,GRB2,PPP2R4,YWHAZ,PRKAR2A,PRKACA, PRKCE
mTOR signaling	2.21E00	7/210 (0.033)	RPS4Y1,RPS3A,RHOB,PPP2R4,RPS18,PRKCE,RPS25
EIF2 signaling	1.65E00	6/200 (0.03)	RPS4Y1,RPS3A,GRB2,RPS18,RPS25,RPLP0

This table corresponds to Figure 1, and list the proteins implicated in mitochondrial dysfunction, CME, 14-3-3 mediated signaling, ERK,/MAPK signaling, mTor, and EIF2 signaling.

### Candidate protein changes in haloperidol treated mice

We examined the hippocampal protein profile from mice chronically treated with haloperidol for the 114 candidate proteins identified to be differentially expressed following n-3 deficiency. We used the same LC-MS/MS platform described above. Twenty-three of the 114 n-3 candidate proteins were identified as differentially expressed in the haloperidol treated animals and of these, 17 of which were significantly altered in the opposite direction to that observed in n-3 deficiency (Supplementary Table S1).

## Discussion

We used label free LC-MS/MS to study the consequences of n-3 FA deficiency on protein expression in the hippocampus of mice. We profiled 1008 proteins for differential expression, and identified 114 proteins as significantly altered between n-3 deficient and control groups. The major biological processes disturbed by n-3 deficiency included endocytosis, neuritogenesis, and exocytosis (Table 3), and the major signaling pathways altered were mitochondrial dysfunction, CME, 14-3-3 signaling (Table 4). Synaptic function was thus significantly implicated as a biological process (endocytosis and exocytosis) and pathway (CME), followed by disturbances in neuritogenesis, mitochondrial dysfunction, and 14-3-3 signaling. As these functions have previously been reported to be abnormal in schizophrenia (Harrison and Weinberger, 2005; English et al., 2011, 2012; Kim et al., 2012; Manji et al., 2012), our data is consistent with the view that n-3 deficiency is an environmental risk factor for schizophrenia (McNamara, 2011).

Synaptic function was significantly disturbed with alternations in both endocytosis (12 proteins) and exocytosis (6 proteins) functions in the hippocampus of n-3 FA deficient mice. More specifically, and in keeping with our hypothesis, the CME protein pathway was implicated, with 6 proteins decreased (↓ GRB2, ↓ PIP5K1C, ↓ APOE, ↓ DNM1L, ↓ PPP3CB, ↓ ARPC5L), and 4 increased (↑ CLTC, ↑ HSPA8, ↑ UBC, ↑ SH3GL2) in expression. CME is the best-characterized endocytotic pathway for cellular membrane and protein trafficking, and post-mortem studies implicate CME as one a potential core pathophysiological processes in schizophrenia (Schubert et al., 2012). Two of the above CME proteins, CLTC (Allen et al., 2008) and HSPA8 (Föcking et al., 2011), have previously been implicated in schizophrenia. In keeping with our data, Ben Gedalya and colleagues have previously shown that n-3 FA augmentation are capable of enhancing CME in neuronal cultured cells (Ben Gedalya et al., 2009). Other endocytosis (↑ RHOB, ↑ MYO5A, ↑ ATP5B, ↓ CAP1), and exocytosis (RAB3A, SRCIN1, RALA) proteins were also significantly altered, along with SNARE protein SNAP 47 (↑2.1 fold). In addition, the post synaptic density (PSD) protein SHANK3 was significantly increased (↑1.68 fold) in expression. SHANK3 is a major susceptibility gene in schizophrenia (Gauthier et al., 2010) and we have recently observed decreased SHANK3 expression in the PSD in schizophrenia (Föcking et al., 2012). As disturbed CME and synaptic processes are core features in the pathophysiology of psychiatric disorders, this study provides further evidence that n-3 FA deficiency is a risk factor for schizophrenia.

Neuritogenesis was the second most significant function implicated following n-3 FA deficiency with 6 proteins significantly decreased (↓SEPT11, ↓PIP5K1C, ↓APOE, ↓VAPA, ↓RALA, ↓DNM1L), and 9 increased (↑RAB3A, ↑STMN1, ↑GJA1, ↑ABI2, ↑RTN3, ↑RTN4, ↑CTNNA2, ↑MYO5A, ↑RHOB) in expression. Seven neuritogenesis proteins have roles in dendritic growth and branching (↓SEPT11, ↓APOE, ↓DNM1L, ↑CTNNA2, ↑MYO5A, ↑RHOB, ↑RTN4) and one of these RTN4 (↑1.76 fold) a potent neurite outgrowth inhibitor (Chen et al., 2000). CTNNA2 (↑1.5 fold) is a schizophrenia susceptibility gene (Chu and Liu, 2010), and functions in dendrite morphogenesis, cell adhesion, and regulation of synaptic structural plasticity and has recently been implicated in a pluripotent stem cell model of schizophrenia (Pedrosa et al., 2011). SEPT11 (↓1.6 fold) has previously been observed to be reduced in the hippocampus in schizophrenia (Föcking et al., 2011). Importantly, six of the neuritogenesis proteins implicated here (SEPT11, RHOB, RALA, DNM1L, RTN4, MYO5A), have expression changes consistent with decreased neuritogenesis (Chen et al., 2000; Rao et al., 2002; Lalli and Hall, 2005; Li et al., 2009; McNair et al., 2010; Dickey and Strack, 2011), suggesting that neuritogenesis is reduced as a consequence of n-3 FA deficiency. These findings are in keeping with previous studies that show enhanced hippocampal neuritogenesis following DHA supplementation (Kawakita et al., 2006; Beltz et al., 2007; He et al., 2009).

Mitochondrial dysfunction is described in Alzheimer's, Huntington's, and Parkinson's disease (Eckert et al., 2013), and there is now increasing evidence implicating mitochondrial dysfunction in schizophrenia and mood disorders (Martins-De-Souza et al., 2011; Manji et al., 2012). Mitochondrial dysfunction occurs when the antioxidant defence system is overpowered by ROS-mediated oxidative stress, triggered by factors such as genetic defects, environmental exposures, and metabolic fluctuations. In the current study, we observed 10 mitochondrial dysfunction proteins to be significantly increased by n-3 FA deficiency (↑ATP5A1, ↑ATP5AB, ↑COX4I1, ↑NDUFA8, ↑NDUFA13, ↑NDUFV1, ↑OGDH, ↑PDHA1, ↑UQCRC1, ↑UQCR10). This uniform and significant up-regulation of mitochondrial dysfunction suggests n-3 FA deficiency is an environmental risk factor (dietary intake) capable of triggering cellular oxidative stress. In addition, mitochondria have important roles in regulating synaptic plasticity, learning, and memory, due to their key roles in processes such as ATP production (Manji et al., 2012), Ca^2+^ homeostasis (Gleichmann and Mattson, 2011), AMPA receptor trafficking in the synaptic membrane (Li et al., 2010), and apoptosis (Youle and Strasser, 2008), the latter of which is particularly important during neurogenesis (Deng et al., 2010). Dysfunctions in these processes are important neuropathological features of neurodegenerative and psychiatric diseases, and indeed 7 of the 10 significant mitochondrial proteins have previously been implicated at the mRNA level in either Alzheimer's, Huntington disease, or schizophrenia (Supplementary Table S1). In addition, ATP5A1 has previously been identified as significantly increased in proteomic studies of post-mortem brain in schizophrenia (English et al., 2011) and bipolar disorder (Pennington et al., 2008). In support of these findings, *in vivo* and *ex vivo* animal studies showed an improvement in mitochondrial function after treatment with n-3 FA (Eckert et al., 2013); and more specifically augmentation with DHA improved mitochondrial ATP generation in the CA1 area of the hippocampus (Harbeby et al., 2012). Moreover, a recent study by Feng and colleagues found that maternal DHA supplementation protects against prenatal stress induced impairment of learning and memory, and normalized mitochondrial, apoptotic, and oxidative stress proteins altered as a consequence of prenatal stress (Feng et al., 2012). The ability of dietary n-3 FA to consistently and uniformly modulate mitochondrial function may thus be instrumental in improving cognitive and behavioral abnormalities, as well as providing neuroprotection in vulnerable diseases states. In addition, 14-3-3 signaling pathway was also increased by deficiency, with 7 proteins up-regulated (↑PRKCE, ↑TUBA1A, ↑TUBA4A, ↑GRB2, ↑YWHAE, ↑YWHAQ, ↑YWHAZ). The 14-3-3 proteins are consistently implicated in schizophrenia proteomic studies (English et al., 2011), but not specifically (Zabel et al., 2008), and have a wide variety of functions including cell cycle control, cellular signaling, and stress response.

The expression of the 114 candidate proteins were examined following chronic treatment with the antipsychotic haloperidol in the hippocampus. Twenty-three of the 114 proteins were significantly altered by haloperidol treatment and of these 17 were differentially expressed in the opposite direction to that observed following n-3 FA deficiency. These included proteins involved in neuritogenesis (↓CTNNA2, ↓STMN1), mitochondrial dysfunction (↓ATP5A1, ↓ATP5B, ↓UQCRC1), CME (↑ARPCL5, ↓HSPA8), ribosomal proteins (↑RPS18, ↑RPS3A), and others (↑ARL3, ↑CASKIN1, ↓GDI2, ↓NSFL1C, ↓PKM2, ↓PRKACA, ↓PYGB) as detailed in Supplementary Table S1. These findings provide preliminary evidence that antipsychotic drug treatment and n-3 FA deficiency share differential expression of several protein, and this evidence provides clues as to the that pathways that may be implicated in disease pathology.

This study has limitations and we acknowledge that the major findings were not validated by traditional methods such as western blotting or ELISA. However, our quantification method was based on triplicate LC-MS/MS runs for each sample, a strategy that was not feasible in previous 2D gel based experiments. In addition, reliability and consistency was confirmed by visual inspection of spectra, trypsin autolysis peaks, and stable LC-MS/MS retention times across runs (data not shown). Total peptide count (no. of peptides identified per protein) were checked for consistency within replicate runs, and across samples to ensure the quality of the data. It should also be noted that our model of deficiency, through 2 generations, maybe considered extreme but previous work in these animals had demonstrated greater learning and cogitative defects in the second generation, as well as alterations in dopamine availability, respectively (Moriguchi et al., 2000; Bondi et al., 2013).

This is the first study to extensively characterize the proteomic and molecular pathways disturbed in the hippocampus as consequence of n-3 FA deficiency. In light of this data, it is conceivable that abnormalities of synaptic function, neuritogenesis, and mitochondrial dysfunction exists in individuals chronically deficient in n-3 FA, and that the modulation of these processes are a mechanisms by which n-3 FA improve symptoms in subjects in the at risk mental state (Amminger et al., 2010). This suggestion is in keeping with findings that neuritogenesis (Kawakita et al., 2006; Beltz et al., 2007; He et al., 2009) and mitochondrial function (Eckert et al., 2013) are significantly improved following n-3 augmentation, including that following prenatal stress (Feng et al., 2012), and these changes coincided with improvements in cognitive and depressive-like behaviors. Future studies will focus on understanding the brain proteomic response to n-3 FA supplementation following deficiency, and indeed by studying sub-proteomes such as membrane proteins (English et al., 2012) in n-3 deficient or n-3 ameliorated states.

### Conflict of interest statement

The authors declare that the research was conducted in the absence of any commercial or financial relationships that could be construed as a potential conflict of interest.

## Supplementary material

Supplementary Table S1 **List of 114 proteins significantly differentially expressed as a consequence of dietary n-3 deficiency in the mouse hippocampus.** The table is ordered *p*-value, and proteins that were significant after permutation based FDR at 5% threshold are marked with an ^*^. Further details on fold change, protein name, and the fold change of proteins that were significant in the haloperidol treated mouse hippocampus are also provided for each protein entry. In addition, IPA information on the most significant canonical pathways and neurological diseases are included.

Supplementary Figure S1 **(A)** Representative MS1 peak which eluted at 12.75minutes, with a m/z of 628.82. The peak width ranged between 12.41 and 12.92 min with at least 15 data points acquired across the 30 s peak. **(B)** Representative MS1 peak which eluted at 19.93 min, with a m/z of 360.72. The peak width ranged between 19.76 and 20.30 min with at least14 data points acquired across the 30 s peak. **(C)** Representative MS1 peak which eluted at 22.86 min, with a m/z of 528.26. The peak width ranged between 22.56 and 23.13 min with at least 23 data points acquired across the 30 s peak.

Supplementary Figure S2 **The top IPA biological functions disrupted as a consequence of n-3 FA deficiency**.
