# Supplementary figures and images for: Omega-3 fatty acid deficiency disrupts endocytosis, neuritogenesis, and mitochondrial protein pathways in the mouse hippocampus
Source: Front Genet. 2013 Oct 28;4:208. doi: 10.3389/fgene.2013.00208 (PMC3809566; doi:10.3389/fgene.2013.00208)

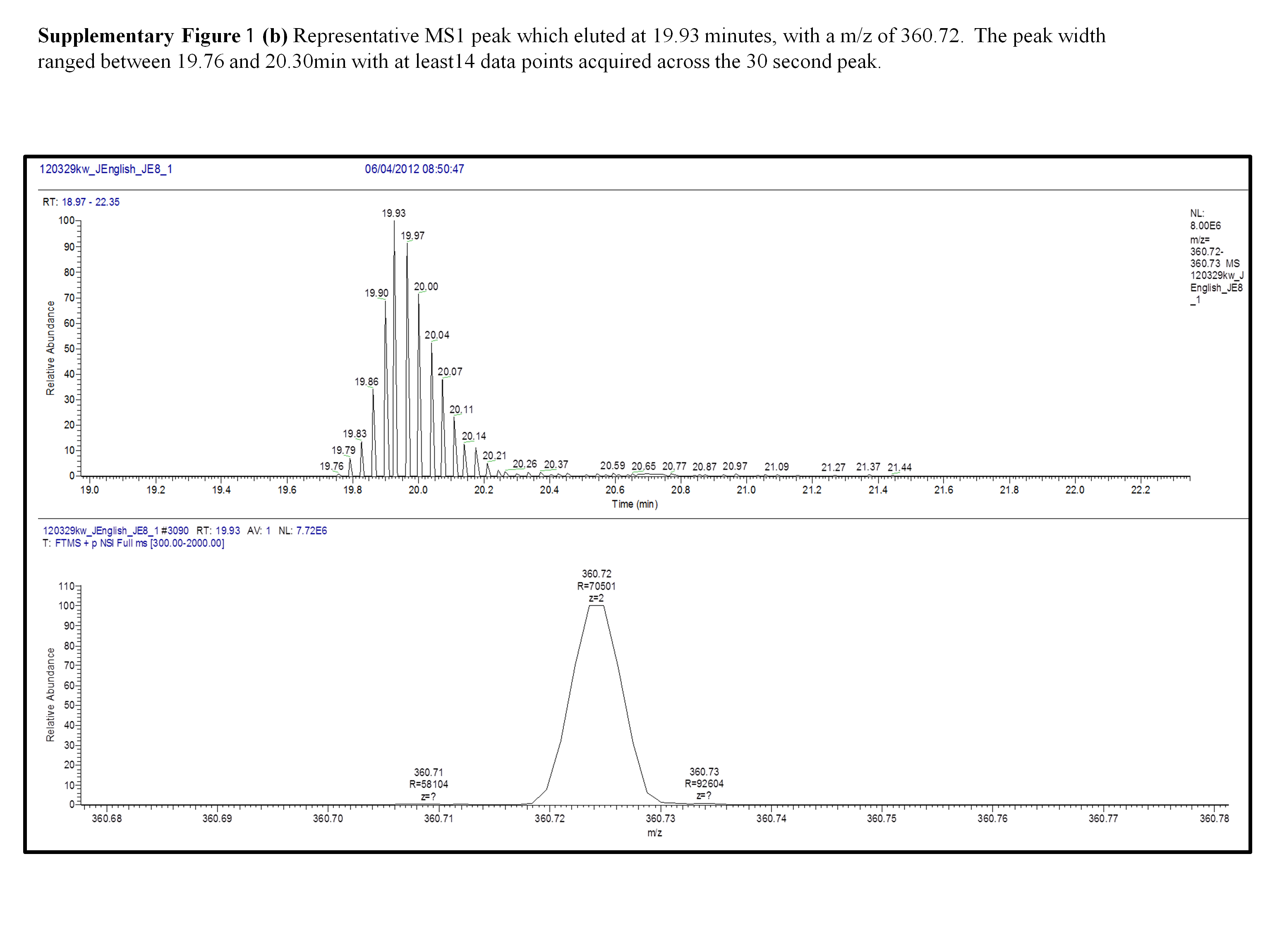

Supplement: Supplementary Figure S1 — (A) Representative MS1 peak which eluted at 12.75minutes, with a m/z of 628.82. The peak width ranged between 12.41 and 12.92 min with at least 15 data points acquired across the 30 s peak. (B) Representative MS1 peak which eluted at 19.93 min, with a m/z of 360.72. The peak width ranged between 19.76 and 20.30 min with at least14 data points acquired across the 30 s peak. (C) Representative MS1 peak which eluted at 22.86 min, with a m/z of 528.26. The peak width ranged between 22.56 and 23.13 min with at least 23 data points acquired across the 30 s peak. [file Presentation1.ZIP › 63730_English_Supp_Figure_1b.tif]

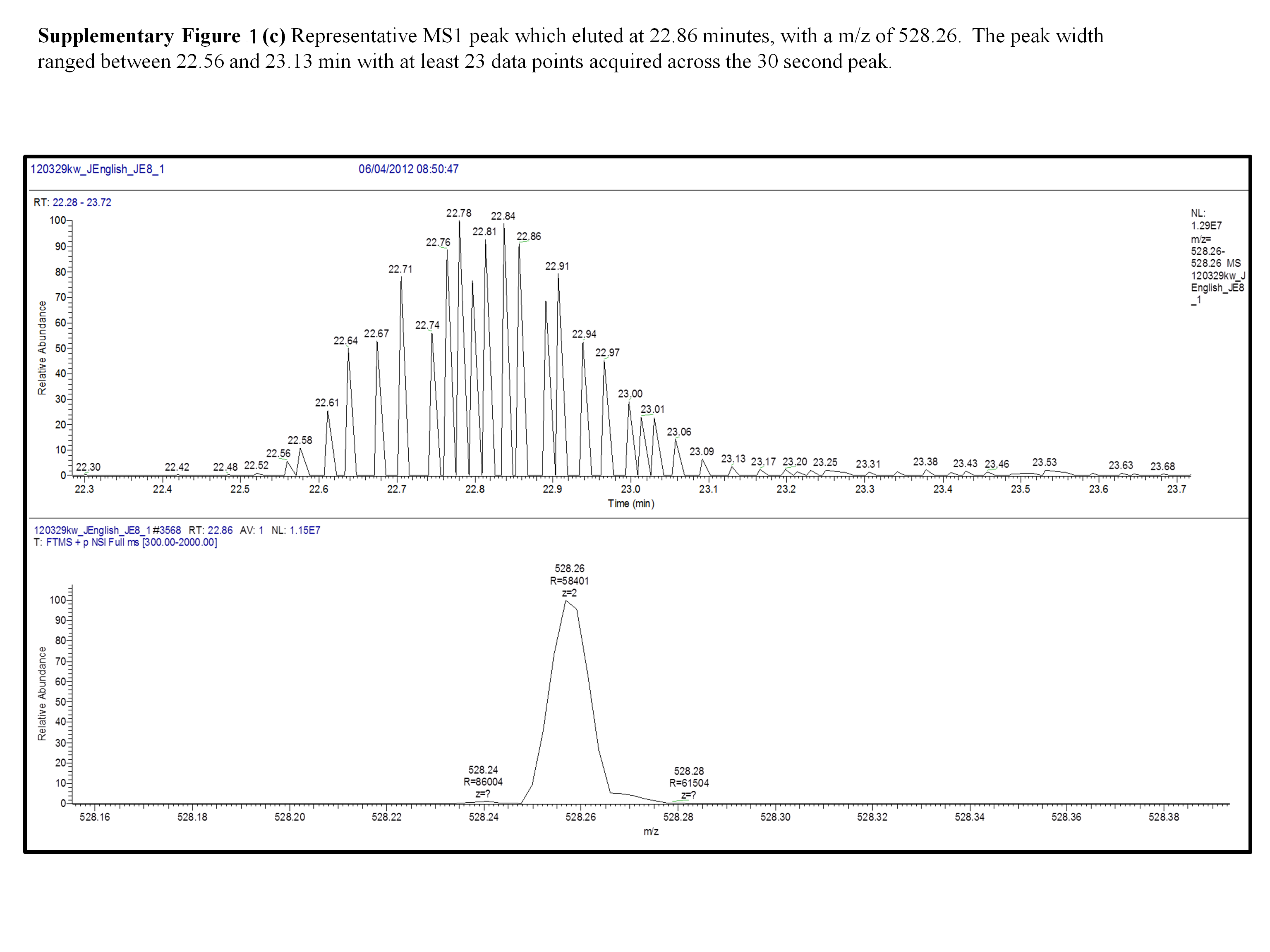

Supplement: Supplementary Figure S1 — (A) Representative MS1 peak which eluted at 12.75minutes, with a m/z of 628.82. The peak width ranged between 12.41 and 12.92 min with at least 15 data points acquired across the 30 s peak. (B) Representative MS1 peak which eluted at 19.93 min, with a m/z of 360.72. The peak width ranged between 19.76 and 20.30 min with at least14 data points acquired across the 30 s peak. (C) Representative MS1 peak which eluted at 22.86 min, with a m/z of 528.26. The peak width ranged between 22.56 and 23.13 min with at least 23 data points acquired across the 30 s peak. [file Presentation1.ZIP › 63730_English_Supp_Figure_1c.tif]

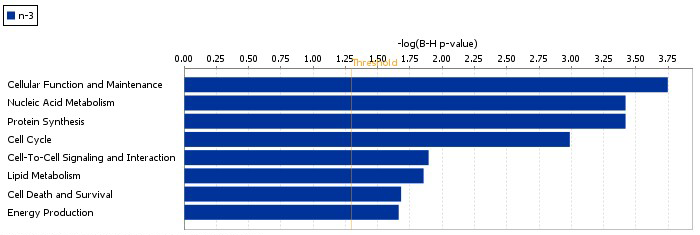

Supplement: Supplementary Figure S1 — (A) Representative MS1 peak which eluted at 12.75minutes, with a m/z of 628.82. The peak width ranged between 12.41 and 12.92 min with at least 15 data points acquired across the 30 s peak. (B) Representative MS1 peak which eluted at 19.93 min, with a m/z of 360.72. The peak width ranged between 19.76 and 20.30 min with at least14 data points acquired across the 30 s peak. (C) Representative MS1 peak which eluted at 22.86 min, with a m/z of 528.26. The peak width ranged between 22.56 and 23.13 min with at least 23 data points acquired across the 30 s peak. [file Presentation1.ZIP › 63730_English_Supp_Figure_2.tif]

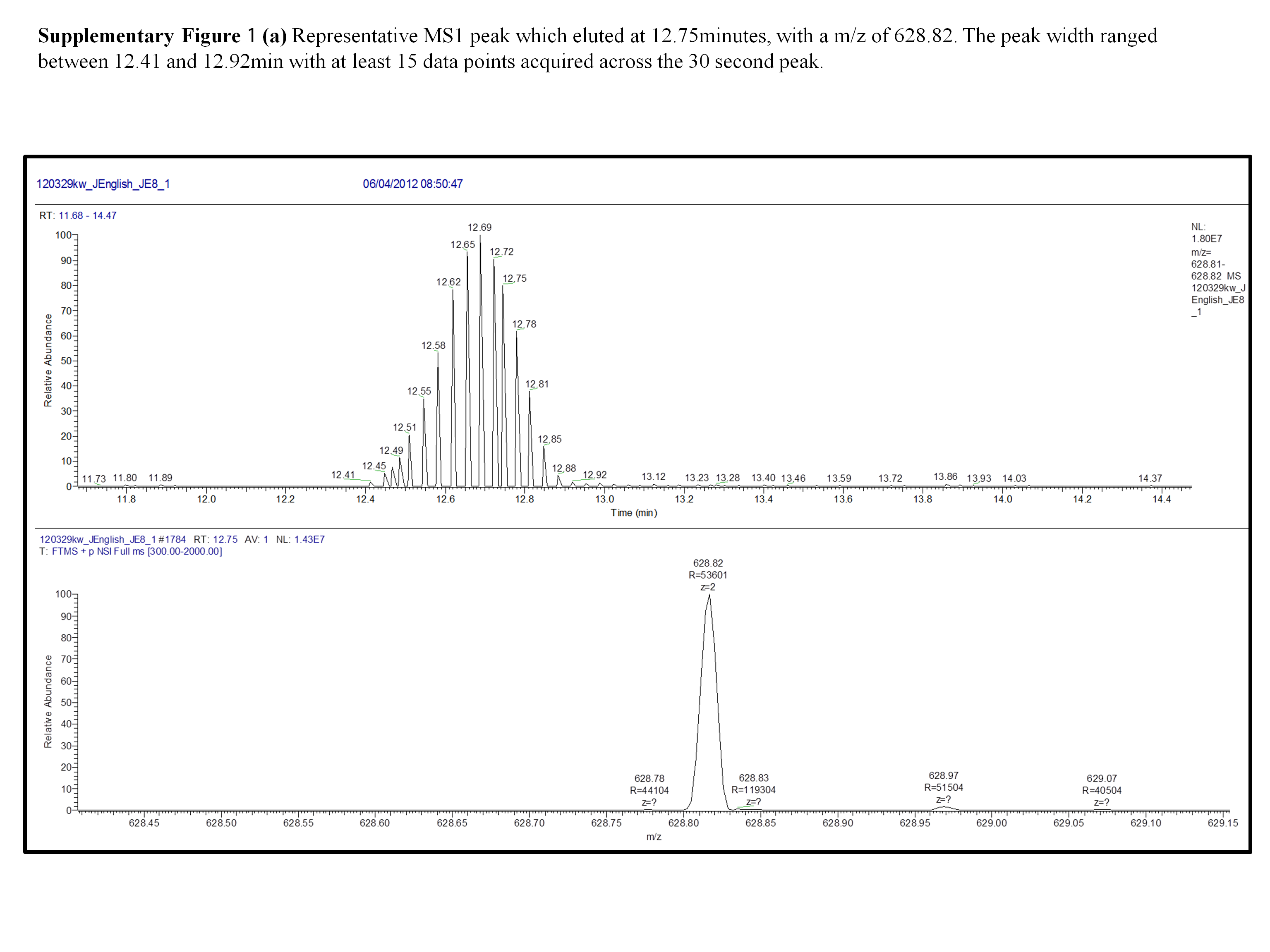

Supplement: Supplementary Figure S1 — (A) Representative MS1 peak which eluted at 12.75minutes, with a m/z of 628.82. The peak width ranged between 12.41 and 12.92 min with at least 15 data points acquired across the 30 s peak. (B) Representative MS1 peak which eluted at 19.93 min, with a m/z of 360.72. The peak width ranged between 19.76 and 20.30 min with at least14 data points acquired across the 30 s peak. (C) Representative MS1 peak which eluted at 22.86 min, with a m/z of 528.26. The peak width ranged between 22.56 and 23.13 min with at least 23 data points acquired across the 30 s peak. [file Presentation1.ZIP › 63730_English_Supp_Figure_1a.tif]
